# Fine needle aspiration cytology guided by ultrasound in the diagnosis of subcentimetre thyroid nodules

**DOI:** 10.1186/s40064-016-2555-0

**Published:** 2016-06-24

**Authors:** Cheng Li, Weiwei Zhan, Fang Yi, Bin Zheng, Yaqin Zhou, Ran Zhao, Yi Jia

**Affiliations:** Department of Ultrasound, North Branch of Ruijin Hospital, Shanghai Jiaotong University School of Medicine, Shanghai, China; Department of Ultrasound, Ruijin Hospital, Shanghai Jiaotong University School of Medicine, Shanghai, China

**Keywords:** Thyroid nodules, Ultrasonography, Ultrasound-guided fine-needle aspiration biopsy, Cytology

## Abstract

**Background:**

This study aimed to investigate the value of fine needle aspiration biopsy (FNAB) under ultrasound guidance in diagnosis of thyroid nodules. In a retrospective analysis of FNAB in 1050 cases of patients with 1100 nodules, patients were divided according to the maximum diameter of their nodules into two groups: >1.0 and ≤1.0 cm. The ultrasound-guided FNAB cytology results were compared between two groups.

**Results:**

Ultrasound findings showed that among 1100 thyroid nodules, 547 were highly suspicious, 358 were moderately, 175 were low, and 19 were very low. Cytology results showed papillary carcinomas in 453, possible papillary cancer in 126 cases, follicular tumors in 26, suspicious follicular tumors in 6, atypical cells in 7, nodular goiter in 289, colloid in 13, chronic lymphocytic thyroiditis in 175, and undiagnosed specimen in 5. Ultrasound diagnosis of thyroid nodules had an overall sensitivity of 86.0 %, and a specificity of 81.9 %. In nodules larger than 1.0 cm, the sensitivity was 92.8 %, and the specificity 92.3 %. In nodules ≤1.0 cm, the sensitivity was 82.4 %, and the specificity was 81.7 %.

**Conclusions:**

Patients with highly suspicious thyroid nodules on ultrasonography, regardless of nodule sizes, should receive ultrasound-guided FNAB to confirm their natures and direct clinical managements.

## Background

With the improved resolution of high frequency ultrasound and new technologies, the detection rate of thyroid nodules has improved significantly, but it is still necessary to use ultrasound-guided fine-needle aspiration biopsy (US-FNAB) to determine the nature of thyroid nodules (Singh Ospina et al. 2016; Kaliszewski et al. 2016). US-FNAB has many advantages, such as real-time guidance, simple operation, safe, few contraindications and complications, and an effective method to identify the benign and malignant thyroid nodules (Cooper et al. 2009; Moon et al. 2012; Kim et al. 2009a, b; Lee et al. 2011). This study was a retrospective analysis of 1050 patients with a total of 1100 thyroid nodules, which were divided into two groups of the maximum diameter over 1.0 cm and less than or equaling to 1.0 cm, Ultrasound and cytology were adopted to analyze the value of US-FNAB in the diagnosis of thyroid nodules.

## Methods

### Study design

US-FNAB results of patients with thyroid nodules from January 2013 to December 2013 were retrospectively analyzed. In 2013, there were more than 40,000 patients underwent thyroid ultrasound in Department of Ultrasound of Ruijin Hospital. Among them, there are 5000 thyroid patients received US-FNAB, and about 4000 patients received surgical interventions. Inclusion criteria were as follows: 1. patients receiving thyroid ultrasound in our hospital; 2. nodules with cytological results; 3. nodules suspicions for malignancy confirmed by surgical pathology; 4. negative cytological nodules confirmed by follow-ups for more than 1 year with unchanged ultrasound appearances or by surgical pathology (Frates et al. 2005). A total of 1050 cases of patients (298 males and 752 females) with a mean age of 46.0 ± 13.5 years old (range 16–84 years old). A total of 1100 thyroid nodules were divided into two groups with the maximum diameter over 1.0 cm and the maximum diameter less than or equaling to 1.0 cm.

### Instruments and methods

A SIEMENS S2000 using color ultrasound scanner with an ACUSON-18L6HD UHF probe was adopted and the instrument was adjusted to the best image quality. Every patient lay in a supine position with a thin pillow under the shoulders, and the neck extended. A comprehensive thyroid scan was performed to detect any nodule and images were stored.

### Ultrasound thyroid nodules and classification criteria

The number, location, size, shape, aspect ratio, margin, ultrasound halo, internal structure, calcifications, posterior echo, and the extent and pattern of the blood supply of nodules were evaluated. The thyroid nodules and differentiated thyroid cancer treatment guidelines released by American Thyroid Association (ATA) in 2014 based on ultrasound characteristics to predict risks of malignancy. The guideline considered sonographic features of malignant nodules as hypoechoic texture with irregular margins (such as surrounding infiltration and small projections), microcalcifications, vertical growth, and nodular or partial annular calcification. Based on these features, thyroid nodules were divided into high, moderate, low, and very low degrees of suspicion, and benign ones. High suspicion referred to solid hypoechoic nodules or a solid hypoechoic nodule with a cystic component, and the combination of the following characteristics: irregular edges (such as invasion of the surrounding tissue), microcalcifications, vertical growth, nodular or partial annular calcification with destruction zone, hypoechoic soft tissue protrusion, invasion into surrounding thyroid. Moderate suspicion referred to hypoechoic solid nodules with smooth margins, no microcalcifications, no invasion, and vertical growth. Low suspicion referred to hyperechoic nodules or cystic nodules, solid areas, uniform texture, no microcalcifications, regular margins, no extracapsular spread, and non-vertical orientation. Very low suspicion referred sponge-like nodules or partially cystic nodules, not associated with low, medium and high suspicion in any one of sonographic features. Benign ones referred to purely cystic nodules (Brito et al. 2014; Horvath et al. 2009; Ito et al. 2007; Tae et al. 2007).

In this study, images were retrospectively analyzed by two double-blinded Sonologists to determine the classification according to the 2014 ATA guidelines. They were classified as high, moderate, low, and very low suspicion and benign. If there was disagreement in the classification, the final classification should be reached by consensus.

### US-FNAB material

The materials consisted of 5 ml disposable plastic syringes, 27G syringe needles, gloves, iodine swabs, 1 % lidocaine, slides, bottles and ethanol fixed fast staining solution.

### US-FNAB specimen collection

Preoperative coagulation function and body condition of all patients were assessed. Informed consent was obtained. After routine disinfection, sterile towels were applied, and lidocaine was injected for local anesthesia to the thyroid capsule under ultrasound guidance. Under ultrasound-guidance, specimens were collected by aspirated into 5 ml plastic syringes. Thyroid specimens were collected 2–6 times, according to the specific circumstances of each case.

### Standard thyroid nodule cytology specimens

There was an on site cytologist in the cell room next to the ultrasound examination room. By using the “Thyroid Cytopathology Bethesda reporting system (Cibas and Ali 2009): definitions, standards and notes”, the specimens were divided into six categories: I: specimen cannot be diagnosed or was unsatisfactory, II: benign, III: equivocal lesions (atypical cells or follicular lesions), IV: follicular neoplasm or suspicious follicular tumors, V: suspicious for malignancy, VI: malignancy. According to the 2010 AACE/AME/ETA “European thyroid nodule diagnosis and treatment guidelines”, Bethesda categories I to IV were classified as “negative cytology”, while categories V and VI were classified as “positive cytology”.

### Statistical analysis

In results of US-FNAB cytology, surgical pathology and ultrasound follow-up of more than 1 year, data were analyzed by using SPSS version 19.0 statistical software. t-test was used to compare the cytological categories between two groups, and numerical data were compared with χ^2^ test, while consistency of paired data was analyzed with Kappa test.

## Results

In the 1050 patients, a total of 1100 nodules of maximum diameters from 0.29 to 2.8 cm with an average diameter of 1.08 ± 0.53 cm were found. There were 322 nodules larger than 1.0 cm and 778 of 1 cm or smaller (amongst which 292 cases were smaller than 0.5 cm). Cytological results showed benign in 521 nodules (confirmed by surgery in 23, and by follow-ups for more than 1 year in 493). Cytological results were malignant in 579 nodules, confirmed by surgery. Cytological results were satisfactory in 1095 (99.55 %) cases and non-diagnostic or unsatisfactory in 5 (0.45 %), all of which were 0.6 cm or smaller and were managed by follow-ups.

### Thyroid nodule ultrasound results

The 2014 ATA guidelines recommend dividing thyroid nodules into five categories based on ultrasonographic features, which were high, moderate, low, very low level of suspicion of malignancy, and benign. Results showed benign in one nodule (0.1 %); very low level of suspicion in 19 nodules (1.7 %); low level of suspicion in 175 nodules (15.9 %); moderate level of suspicion in 358 nodules (32.5 %); and high level of suspicion in 547 nodules (49.7 %). Among nodules smaller than 1.0 cm, results showed benign in 0 nodules (0 %); very low level of suspicion in 8 nodules (1 %); low level of suspicion in 98 nodules (12.6 %); moderate level of suspicion in 254 nodules (32.6 %); and high level of suspicion in 418 nodules (53.7 %). Among those larger than 1.0 cm, results showed benign in 1 nodule (0.3 %); very low level of suspicion in 11 nodules (3.4 %); low level of suspicion in 77 nodules (23.9 %); moderate level of suspicion in 104 nodules (32.3 %); and high level of suspicion in 129 nodules (40.1 %).

### US-FNAB cytology results

The overall US-FNAB cytology results are shown in Table 1. US-FNAB cytopathological results of this study, and the results according to nodule sizes are shown in Table 2.

**Table 1:** Bethesda cytology of thyroid nodules

Cytology pathology	Nodules	Ratio (%)
I	5	0.5
II	477	43.4
III	7	0.6
IV	32	2.9
V	126	11.5
VI	453	41.2
Total	1100	100.0

**Table 2:** US-FNAB cytology pathology Bethesda results by nodule size

Nodule size	I	II	III	IV	V	VI	Total
Maximum diameter ≤1.0 cm	3	311	6	20	95	343	778
Maximum diameter >1.0 cm	2	166	1	12	31	110	322
Total	5	477	7	32	126	453	1100

χ^2^ = 15.900, p = 0.007

### Ultrasound compared with US-FNAB cytology results

The relationship between ultrasound features of nodules and US-FNAB cytopathological is presented in Table 3. The relationship between ultrasound features by nodule sizes and US-FNAB cytology is presented in Table 4, while the effects of the patient’s gender and cytopathological results are presented in Table 5. In this study, the overall sensitivity of ultrasound in diagnosing nodules was 86.0 % and the specificity was 81.9 %. The sensitivity for larger nodules was 92.8 %, and the specificity was 92.3 %, while for smaller nodules the sensitivity was 82.4 %, and the specificity was 81.7 %.

**Table 3:** Ultrasound findings vs US-FNAB cytology Results

Ultrasound	Negative	Positive	Total
Benign	1	0	1
Very low suspicion	18	1	19
Low suspicion	133	42	175
Moderate suspicion	296	62	358
High suspicion	73	474	547
Total	521	579	1100

**Table 4:** US-FNAB cytology results by lesion size

	Ultrasound	Negative	Positive
Maximum diameter ≤1.0 cm^a^	Benign	1	0
	Very low suspicion	7	1
	Low suspicion	69	29
	Moderate suspicion	204	50
	High suspicion	60	358
	Total	340	438
Maximum diameter >1.0 cm^b^	Benign	1	0
	Very low suspicion	11	0
	Low suspicion	64	13
	Moderate suspicion	92	12
	High suspicion	13	116
	Total	181	141

^a^χ^2^ = 319.369, p < 0.001

^b^χ^2^ = 187.482, p < 0.001

**Table 5:** Cytology by gender and nodule size

	Pathological diagnosis	Male	Female
Maximum diameter ≤1.0 cm^a^	Benign	81	259
	Malignant	128	310
	Total	209	569
Maximum diameter >1.0 cm^b^	Benign	47	134
	Malignant	50	91
	Total	97	225
Totals^c^	Benign	128	393
	Malignant	178	401
	Total	306	794

^a^χ^2^ = 2.841, p = 0.092

^b^χ^2^ = 3.394, p = 0.065

^c^χ^2^ = 5.207, p = 0.022

## Discussion

Thyroid nodules are common abnormalities of the endocrine system. Although the incidence in different regions of the world is different, the overall upward trend is strong (Xu et al. 2015). With the use of high-resolution ultrasound and the introduction of new technologies, such as elastography, ultrasound has become the first choice for the diagnosis of thyroid nodules (Burch et al. 2016). As shown in a national large-scale study, ultrasound examination of the thyroid revealed nodules in 20–76 % (Gharib et al. 2010), which was a dramatic increase over the last 10 years. With a prevalence rate of 5–15 % (Cooper et al. 2009), thyroid cancer has become a common clinical malignancy with an increasing incidence all over the world (Londero et al. 2013; Davies and Welch 2006). The rate of unnecessary thyroid surgery has also increased. US-FNAB is a non-invasive way to discriminate benign thyroid nodules from malignant ones, and can reduce unnecessary diagnostic surgeries.

The 2014 ATA guideline (Horvath et al. 2009; Ito et al. 2007; Tae et al. 2007), relies on sonographic features to predict risks of malignant thyroid nodules, divides them into high, moderate, low, and very low degree of suspicion, and gives the corresponding probability of malignancy. The risk of malignancy in 70–90 % in a thyroid nodule was rated as high suspicion, a risk of malignancy in 10–20 % was rated as moderate suspicion, a risk of malignancy in 5–10 % was rated as low suspicion, a risk of malignancy in <3 % was rated as very low suspicion, and a risk of malignancy in <1 % was rated as benign nodules. Results of this study showed that in nodules rated as “high” and “moderate” suspicion, the proportion of thyroid nodules with positive cytological results was similar to the 2014 ATA guidelines. For nodules with “low”, and “very low” degrees of suspicion, the rate of positive US-FNAB cytological results was significantly higher than that in the guidelines. Management of thyroid nodules depends on the level of suspicion. For nodules with high and moderate suspicion, US-FNAB is recommended, while follow-up is generally recommended for nodules with low degree of suspicion and US-FNAB is not needed unless there is a family history of thyroid cancer, exposure to ionizing radiation or a strong biopsy request from patients. When the level of suspicion is very low, US-FNABs is not needed.

The 2014 ATA guideline stated that US-FNAB was the most reliable method for preliminary diagnosis of thyroid nodules (Cooper et al. 2009). Many literatures (Nayar and Ivanovic 2009; Bongiovanni et al. 2012; Luu et al. 2011; Theoharis et al. 2009) described patients in whom US-FNAB was done in 89–95 % of cases, including 55–74 % cases diagnosed as benign, 2–5 % diagnosed as malignant, and the remaining diagnosed as “indeterminate cytology”. The nodules with unsatisfactory biopsies (Moon et al. 2012; Horvath et al. 2009; Chen et al. 2009) mainly include cystic nodules, calcified nodules, nodules with a rich blood supply, and tough nodules difficult to be punctured. Nodules smaller than 6 mm and benign nodules are likely to yield insufficient amount of cells. The question of how to deal with cases when the cytology is not clear is an active area of research. For cases with indeterminate cytological results, ATA guidelines recommended a repeat biopsy. From the literature of Cooper, a repeat biopsy can confirm the diagnosis in about 75 % of solid nodules (Cooper et al. 2009). In this study, it was able to obtain satisfactory specimens in 99.55 % cases, which was higher than that reported in the literature. In addition to operator’s experience, it was suggested that it was not easy to obtain a satisfactory specimen if less than six punctures were made (Kim et al. 2009a).

The relevance of nodule size to the results of US-FNAB has been controversial. The 2006 ATA guidelines recommended US-FNAB for nodules with diameters larger than 1.0 cm, the 2009 edition reduced the size of suspicious nodules to a diameter larger than 0.5 cm, and the 2014 edition was skeptical about the value of biopsy for nodules with diameters less than 1.0 cm. Recommendations for US-FNAB of nodules with diameters larger than 1.0 cm should be based on the following criteria: findings outside the thyroid such as invasion or lymph node metastasis, the patient’s age, and the demands of process (Frates et al. 2005; Mazzaferri and Sipos 2008). However, some scholars (Kim et al. 2009a, b; Baskin and Duick 2006) believed that early diagnosis of thyroid cancer could reduce the recurrence rate and mortality regardless of nodule sizes. Long-term follow-up of small biopsy-proven papillary thyroid cancers did not show distant metastases or mortality (Mazzaferri and Sipos 2008), but young patients (younger than 40 years old) were prone to clinical progression, including rapid tumor growth and cervical lymph node metastases. From the histological point of view, the literatures (Nikiforov and Ohori 2012; Ghossein et al. 2007; Morris et al. 2010) on papillary thyroid carcinoma listed a dozen subtypes, some of which carried a poor prognosis with a high recurrence rate, and a low tumor-related survival rate. In these cases, the patient’s age, tumor size and clinical staging were irrelevant. Therefore, regardless of the size of thyroid nodules, clarifying their nature to guide clinical management still has a great value.

In this study, the results of US-FNAB cytology of thyroid nodules with diameters of 1.0 cm or smaller was similar to those larger than 1.0 cm. However, in 182 patients younger than 40 years old with highly suspicious thyroid nodules, US-FNAB cytology was positive in 179 (98.4 %). Thus, for highly suspicious thyroid nodules, especially in patients younger than 40 years old, timely US-FNAB is clinically significant for confirmation of their natures.

However, this study had some limitations. This study was a retrospective study. The majority of patients with benign cytology were followed up, since thyroid cancer develops slowly. A relatively short follow-up of 1 year did not exclude false negative results. Two cases with positive preoperative US-FNAB but negative histology in very small nodules were excluded.

## Conclusions

Highly suspicious thyroid nodules on ultrasonography, regardless of nodule sizes, should receive US-FNAB to confirm their natures and direct clinical managements.

## Abbreviation

US-FNAB: ultrasound-guided fine-needle aspiration biopsy
